# Multinational Prospective Cohort Study of Mortality Risk Factors in 198 ICUs of 12 Latin American Countries over 24 Years: The Effects of Healthcare-Associated Infections

**DOI:** 10.1007/s44197-022-00069-x

**Published:** 2022-10-05

**Authors:** Victor Daniel Rosenthal, Ruijie Yin, Sandra Liliana Valderrama-Beltran, Sandra Milena Gualtero, Claudia Yaneth Linares, Guadalupe Aguirre-Avalos, Julio Cesar Mijangos-Méndez, Miguel Ángel Ibarra-Estrada, Luisa Fernanda Jimenez-Alvarez, Lidia Patricia Reyes, Carlos Arturo Alvarez-Moreno, Maria Adelia Zuniga-Chavarria, Ana Marcela Quesada-Mora, Katherine Gomez, Johana Alarcon, Jose Millan Oñate, Daisy Aguilar-De-Moros, Elizabeth Castaño-Guerra, Judith Córdoba, Alejandro Sassoe-Gonzalez, Claudia Marisol Millán-Castillo, Lissette Leyva Xotlanihua, Lina Alejandra Aguilar-Moreno, Juan Sebastian Bravo Ojeda, Ivan Felipe Gutierrez Tobar, Mary Cruz Aleman-Bocanegra, Clara Veronica Echazarreta-Martínez, Belinda Mireya Flores-Sánchez, Yuliana Andrea Cano-Medina, Edwin Giovannny Chapeta-Parada, Rafael Antonio Gonzalez-Niño, Maria Isabel Villegas-Mota, Mildred Montoya-Malváez, Miguel Ángel Cortés-Vázquez, Eduardo Alexandrino Medeiros, Dayana Fram, Daniela Vieira-Escudero, Zhilin Jin

**Affiliations:** 1 Department of Public Health Sciences, University of Miami Miller School of Medicine, Miami, FL USA; 2 Pontificia Universidad Javeriana Hospital Universitario San Ignacio, Bogota, Colombia; 3 Hospital Civil de Guadalajara Fray Antonio Alcalde, Centro Universitario de Ciencias de la Salud, Universidad de Guadalajara, Guadalajara, Mexico; 4 Clinica Universitaria Colombia, Bogota, Colombia; 5 Hospital Clinica Biblica, San José, Costa Rica; 6 Clinica Sebastian de Belalcazar, Cali, Colombia; 7 Hospital del Nino Dr Jose Renan Esquivel, Panama, Panama; 8 Hospital Regional de Alta Especialidad Ixtapaluca, Ixtapaluca, Mexico; 9 Clinica Infantil Santa Maria del Lago, Bogota, Colombia; 10 Hospital San Jose TecSalud, Monterrey, Nuevo Leon, Mexico; 11 Instituto del Corazon de Bucaramanga Sede Bogota, Bogota, Colombia; 12 Instituto Nacional de Perinatologia, Mexico City, Mexico; 13 Hospital Sao Paulo, Universidade Federal de Sao Paulo, Sao Paulo, Brazil; 14 INICC Foundation, International Nosocomial Infection Control Consortium, Miami, USA

**Keywords:** Risk factors, Mortality, Intensive care unit, Healthcare-associated infection, Hospital infection

## Abstract

**Background:**

The International Nosocomial Infection Control Consortium (INICC) has found a high ICU mortality rate in Latin America.

**Methods:**

A prospective cohort study in 198 ICUs of 96 hospitals in 46 cities in 12 Latin American countries to identify mortality risk factors (RF), and data were analyzed using multiple logistic regression.

**Results:**

Between 07/01/1998 and 02/12/2022, 71,685 patients, followed during 652,167 patient-days, acquired 4700 HAIs, and 10,890 died. We prospectively collected data of 16 variables. Following 11 independent mortality RFs were identified in multiple logistic regression: ventilator-associated pneumonia (VAP) acquisition (adjusted odds ratio [aOR] = 1.17; 95% CI: 1.06–1.30; *p* < 0.0001); catheter-associated urinary tract infection (CAUTI) acquisition (aOR = 1.34; 95% CI: 1.15–1.56; *p* < 0.0001); older age, rising risk 2% yearly (aOR = 1.02; 95% CI: 1.01–1.02; *p* < 0.0001); longer indwelling central line(CL)-days, rising risk 3% daily (aOR = 1.03; 95% CI: 1.02–1.03; *p* < 0.0001); longer indwelling urinary catheter(UC)-days, rising risk 1% daily (aOR = 1.01; 95% CI: 1.01–1.26; *p* < 0.0001); higher mechanical ventilation (MV) (aOR = 6.47; 95% CI: 5.96–7.03; *p* < 0.0001) and urinary catheter-utilization ratio (aOR = 1.19; 95% CI: 1.11–1.27; *p* < 0.0001); lower-middle level income country (aOR = 2.94; 95% CI: 2.10–4.12; *p* < 0.0001); private (aOR = 1.50; 95% CI: 1.27–1.77; *p* < 0.0001) or public hospital (aOR = 1.47; 95% CI: 1.24–1.74; *p* < 0.0001) compared with university hospitals; medical hospitalization instead of surgical (aOR = 1.67; 95% CI: 1.59–1.75; *p* < 0.0001); neurologic ICU (aOR = 4.48; 95% CI: 2.68–7.50; *p* < 0.0001); adult oncology ICU (aOR = 3.48; 95% CI: 2.14–5.65; *p* < 0.0001); and others.

**Conclusion:**

Some of the identified mortality RFs are unlikely to change, such as the income level of the country, facility ownership, hospitalization type, ICU type, and age. But some of the mortality RFs we found can be changed, and efforts should be made to reduce CL-days, UC-days, MV-utilization ratio, UC-utilization ratio, and lower VAPs and CAUTI rates.

## Introduction

The goals of medicine include alleviating pain and suffering, promoting health, preventing illness and death, promoting a peaceful death, whenever it is possible to cure the disease, when it is not possible to cure, and avoiding the suffering of the dying [1]. The International Nosocomial Infection Control Consortium (INICC) joins these objectives, promoting throughout the world, the prevention of the acquisition of healthcare-associated infections (HAIs), the suffering that accompanies them, and their adverse consequences [2]. It is the first and biggest multinational HAI research network, established initially in Argentina in 1998 and working internationally since 2002 [2].

Globally, the first HAI rate report was published in 1975 by the US Centers for Disease Control and Prevention (CDC), and for this activity, they have used standardized methods and definitions [3]. INICC has published its first report on HAIs and their associated length of stay (LOS) and mortality in 2006 [4], followed by its reports published in 2008 [5], 2010 [6], 2012 [7], 2014 [8], 2016 [9], 2019 [10], and 2021 [11].

Based on the INICC findings, it has been shown that HAI rates in Latin America [12–19] and in other low- and middle-income countries (LMIC) [4–11] are 3 to 5 times higher than in the US [20]. INICC published that the mortality rate in ICU patients in LMICs including Latin America, without HAI it is 17.12% (95% CI = 16.93–17.32), for those with one HAI is 30.15% (95% CI = 27.70–32.77) to 48.21% (95% CI = 45.57–50.96), and for those with 3 simultaneous HAIs it is 63.44% (95% CI = 55.99–71.60) [11].

Previous researches found that the following are risk factors for mortality in ICU: male gender [21], older age [22], higher APACHE II score [22], SAPS II severity illness score [23], pneumonia [22], blood transfusion [22], immunosuppressive drugs [22], presence of central-line (CL) [22], ventilator-associated pneumonia (VAP) acquisition [22], mechanical ventilation (MV) [24], vasopressors [24], neurological disease [24], low hemoglobin concentration [24], airway disease [24], and others.

Those investigations have found several mortality RFs. However, none of them has analyzed several countries simultaneously aiming to identify mortality RFs in ICUs, and also, nor has any prospective study been conducted for 24 years. So far, we have not found a study that used multiple logistic regression to look at all of the following 16 variables simultaneously and see how they are independently linked to death: (1) Gender, (2) age, (3) type of hospitalization (medical, surgical), (4) in ICU LOS, (5) CL-days, (6) MV-days, (7) UC-days, (8) CL-utilization ratio as a marker of severity of illness of patients, (9) MV-utilization ratio as a marker of severity of illness of patients, (10) UC-utilization ratio as a marker of severity of illness of patients, (11) type of ICU (medical-surgical, medical, pediatric, surgical, coronary, neuro-surgical, cardio-thoracic, neurologic, trauma, pediatric-oncology, adult-oncology), (12) facility ownership (publicly owned facilities, not-for-profit privately owned facilities, for-profit privately owned facilities, university hospitals) [25], (13) income-level per country according to World Bank (WB) (low, lower-middle, upper-middle, high) [26], (14) central line associated bloodstream infection (CLABSI) acquisition, (15) VAP acquisition, and (16) catheter-associated urinary tract infection (CAUTI) acquisition. The present study aims to analyze the independent association of these above enumerated 16 variables as risk factors for in ICU all-cause mortality.

## Methods

### Study Population and Design

This prospective observational cohort study was performed on patients admitted to 198 ICUs of 96 hospitals in 46 cities in 12 countries of Latin America, throughout 24 years, between 07/01/1998 and 02/12/2022, over 24 years.

### Prospective Cohort in ICUs and Surveillance of Health Care-Associated Infections

Each patient’s data were gathered at the time of ICU admission. Infection prevention professionals (IPP) visited each patient’s bedside daily from the time of admission until discharge. This analysis prospectively included all adult and pediatric patients hospitalized to an ICU with or without HAIs, and their data were gathered utilizing the INICC Surveillance Online System (ISOS) [2]. IPPs bring a tablet to each hospitalized patient’s bedside in the ICU, sign in to ISOS, and simultaneously upload the patient’s data [2].

This information is provided at the time of admission and includes information about the setting, such as the nation, city, name of the hospital, and the ICU type, as well as information about the patient, such as age, type of hospitalization, use of invasive devices (CL, MV, UC), and presence of infection [2]. Every day up until the patient is discharged, IPPs upload details on invasive devices (CL, MV, UC), and positive cultures (blood, urine, and respiratory samples) for each patient [2].

If the patient has signs or symptoms of infection, an infectious diseases specialist approach the patient to determine the presence of an HAI (CLABSI, VAP, CAUTI). According to the Centers for Disease Control and Prevention/National Healthcare Safety Network (CDC/NHSN), IPPs look at a patient’s signs and symptoms, cultures, X-rays, and other described criteria to fulfill definitions of HAI [3].

Over the 24 years of this study, all IPPs of all participant hospitals have been applying the current CDC definition of HAIs. That is, whenever the CDC updated their definitions, our IPPs began using the new updated definitions.

When IPPs upload the results of a culture to the ISOS, the ISOS immediately displays a message and directs the IPP to an online module of the ISOS where the IPP can check all the CDC NHSN criteria to determine the presence of a HAI and the kind of HAI (CLABSI, VAE, CAUTI) [2].

Daily device utilization checks are performed by ISOS. When a bias in patient-days or device use is detected from admission to discharge, the ISOS notifies the IPPs. The patient will be hospitalized in the ICU without any devices in place most likely because IPP forgot to upload to ISOS the use of devices or forgot to upload to ISOS the discharge of the patient. If ISOS notices lack of use of any kind of device on any given day, it will send a message to the IPP to remind him or her to upload missing devices or upload the discharge of the patient. In other words, ISOS asks IPPs to look into why a patient in an ICU doesn't have any devices in place [2]. This approach significantly reduces biases associated with device utilization, patient-days, and discharge conditions [2].

Patients with missing data were excluded from this study. The Institutional Review Boards of the participating hospitals provided their approval for this study. Patients' and hospitals' identities are treated with confidentiality.

### INICC Surveillance Online System

Standard CDC/NSHN methodologies state that HAI denominators are device-days gathered from all patients as pooled data, without mentioning the characteristics of particular patients or the quantity of device-days associated with particular patients [3]. INICC HAI surveillance is carried out through the use of an online platform, the INICC Surveillance Online System (ISOS), which includes CDC NHSN criteria and methods [3].

Additionally, ISOS includes the gathering of patient-specific information on all patients, including those with and those without HAI, with a several variables per patient [2]. The ability to match data from all patients admitted to the ICU by different variables allows for the estimation of the mortality RFs.

The CDC/NHSN criteria and methods are used in the data uploaded to ISOS to identify HAIs, estimate HAI rates, and determine device utilization (DU)-ratio [3].

### Validation of Diagnosis of Health Care-Associated Infections

Validation of HAI is a unique feature of the ISOS and is considered essential for maximizing the sensitivity and accuracy of surveillance data. Each HAI reported by an IPP is validated, that is, scrutinized to be certain that criteria are fulfilled to justify its recording as an HAI. All necessary corrections and additions are indicated with a clear red sign on the screen. The validation process also includes the scrutiny of data reported for putatively uninfected patients to permit detection of unreported but true HAI. To accomplish this, when the IPP uploads a culture to the ISOS but does not confirm a HAI, based on the uploaded culture, the date that the culture was taken, and the result of the culture, the ISOS automatic validation system shows an online message to the IPP asking to check CDC/NHSN criteria for that putative HAI, should the ISOS suspect a HAI. Also, the ISOS sends an XLS file to the IPP every month with a list of biases about HAIs that have not been confirmed [2].

### Study Definitions

#### World Bank Country Classifications by Income Level

The WB assigns the world’s economies to four income groups—low, lower-middle, upper-middle, and high-income countries. The classifications are based on gross national income (GNI) per capita in current USD. Low income are those countries with GNI less than USD 1,045. Lower-middle income those with GNI from 1046 to 4095. Upper-middle income those with GNI from 4096 to 12,695. High income those with GNI > 12,695 [26].

#### Facility/Institution Ownership Type

Publicly owned facilities owned or controlled by a governmental unit or another public corporation (where control is defined as the ability to determine the general corporate policy); *not-for-profit privately owned* facilities that are legal or social entities created for the purpose of producing goods and services, whose status does not permit them to be a source of income, profit or other financial gain for the unit(s) that establish, control or finance them; and, *for-profit privately owned facilities* that are legal entities set up for the purpose of producing goods and services and are capable of generating a profit or other financial gain for their owners [25].

#### Patient-Day

A count of the number of patients in a patient care location during a defined time period [27].

#### Device-Utilization

DU was calculated as a ratio of device-days to patient-days for each location type. As such, the DU of a location measures the use of invasive devices and constitutes an extrinsic HAI RF. DU also serve as a marker for severity of illness of patients (i.e., severely ill patients are more likely to require an invasive device) which is an intrinsic HAI RF [28].

#### Ventilator

Any device used to support, assist, or control respiration through the application of positive pressure to the airway when delivered via an artificial airway, specifically an oral/nasal endotracheal or tracheostomy tube.

Definitions of HAI (VAP, CLABSI, CAUTI) used during surveillance were those published by CDC in 1991 [29] and their subsequent updates through 2022 [27].

#### Ventilator-Associated Pneumonia (VAP)

A pneumonia where the patient is on MV for > 2 consecutive calendar days on the date of event, with day of ventilator placement being Day 1, AND the ventilator was in place on the date of event or the day before [27].

#### Clinically Defined Pneumonia

Two or more serial chest imaging test results with at least one of the following: New and persistent or Progressive and persistent; Infiltrate; Consolidation; Cavitation; Pneumatoceles, in infants ≤ 1 year old. For ANY PATIENT, at least one of the following: Fever; Leukopenia; or leukocytosis; For adults ≥ 70 years old, altered mental status with no other recognized cause. And at least two of the following: new onset of purulent sputum or change in character of sputum, or increased respiratory secretions, or increased suctioning requirements; New onset or worsening cough, or dyspnea, or tachypnea; Rales or bronchial breath sounds; Worsening gas exchange; increased oxygen requirements; or increased ventilator demand [27].

#### Pneumonia with Common Bacterial or Filamentous Fungal Pathogens and Specific Laboratory Findings

Two or more serial chest imaging test results with at least one of the following: new and persistent or progressive and persistent Infiltrate; Consolidation; Cavitation; Pneumatoceles, in infants ≤ 1 year old. At least one of the following: fever; leukopenia or leukocytosis; for adults ≥ 70 years old, altered mental status with no other recognized cause. And at least one of the following: new onset of purulent sputum or change in character of sputum, or increased respiratory secretions, or increased suctioning requirements; new onset or worsening cough, or dyspnea, or tachypnea; rales or bronchial breath sounds; worsening gas exchange; increased oxygen requirements; or increased ventilator demand. at least one of the following: organism identified from blood; organism identified from pleural fluid; positive quantitative culture or corresponding semi-quantitative culture result from minimally contaminated LRT specimen; ≥ 5% BAL-obtained cells contain intracellular bacteria on direct microscopic exam; positive quantitative culture or corresponding semi-quantitative culture result of lung tissue; histopathologic exam shows evidences of pneumonia [27].

#### Central Line

An intravascular catheter that terminates at or close to the heart, or in one of the great vessels AND is used for infusion, withdrawal of blood, or hemodynamic monitoring. The following are considered great vessels: aorta, pulmonary artery, superior or inferior vena cava, brachiocephalic veins, internal jugular veins, subclavian veins, external iliac veins, common iliac veins, femoral veins, in neonates, the umbilical artery/vein [3].

#### Primary Bloodstream Infection (BSI)

A Laboratory Confirmed Bloodstream Infection (LCBI) that is not secondary to an infection at another body site [3].

#### Central Line-Associated Bloodstream Infection

A LCBI where an eligible BSI organism is identified, and an eligible CL is present on the LCBI or the day before [3].

#### Laboratory-Confirmed Bloodstream Infection 1

Patient of any age has a recognized bacterial or fungal pathogen, not included on the NHSN common commensal list: identified from one or more blood specimens obtained by a culture OR Identified to the genus or species level by non-culture based microbiologic testing methods. AND Organism(s) identified in the blood are not related to an infection at another site [3].

#### Laboratory-Confirmed Bloodstream Infection 2

A patient of any age has at least *one* of the following signs or symptoms: fever (> 38.0 °C), chills, or hypotension. AND Organism(s) identified in the blood are not related to an infection at another site. AND The same NHSN common commensal is identified by culture from two or more blood specimens collected on separate occasions [3].

#### Common Commensal

Common Commensal organisms include, but are not limited to, *diphtheroids* (*Corynebacterium* spp. not *C. diphtheria*), *Bacillus* spp. (not *B. anthracis*), *Propionibacterium* spp., coagulase-negative *staphylococci* (including *S. epidermidis*), viridans group *streptococci*, *Aerococcus* spp. *Micrococcus* spp. and *Rhodococcus* spp [3].

### Statistical Analysis

Multiple logistic regression was used to compare patients who were alive and those who passed away. Independently, statistically significant factors were associated with mortality. The Wald test was employed as the test statistic, and a level of statistical significance of 0.05 was established. Adjusted odds ratios (aORs) and the corresponding 95% confidence intervals (CIs) for statistically significant variables were calculated from the outputs of multiple logistic regression. The DU-ratio served as a measure of severity of illness. With all the confounders taken into account, we estimated variables that were independently linked with the outcome (in ICU all-cause mortality).

We estimated variables independently associated with the outcome (in ICU all-cause mortality), adjusted to the following prospectively collected data: (1) Gender, (2) age, (3) type of ICU, (4) LOS, (5) CL-days, (6) MV-days, (7) UC-days, (8) CL-utilization ratio, (9) MV-utilization ratio, (10) UC-utilization ratio, (11) type of ICU, (12) facility ownership [25], (13) income-level per country according to WB [26], (14) CLABSI acquisition, (15) VAP acquisition, and (16) CAUTI acquisition. The evaluated outcome was the “in ICU all-cause mortality”. All statistical analyses were performed using R software, version 4.1.3.

## Results

From 07/01/1998 to 02/12/2022, over 24 years, a multinational, multicenter, cohort, prospective, surveillance study of HAIs was conducted in 198 ICUs of 96 hospitals in 46 cities in 12 Latin American countries (Argentina, Brazil, Colombia, Costa Rica, Cuba, Dominican Republic, Ecuador, El Salvador, Mexico, Panama, Peru, Venezuela) currently participating in INICC.

This is a cohort study, and the length of participation of hospitals is variable, and ranged from 1.17 to 227.53 months (mean, 35.02, SD, 42.32). Table 1 show data on facility ownership, ICU type, and other participating hospitals and patients’ characteristics.

**Table 1:** Setting and patient characteristics

Period	1998-07-01 to 2022-02-10
Years, *n*	24
ICUs, *n*	198
Hospitals, *n* (%)	96
Cities, *n* (%)	46
Countries, *n* (%)	12
Total patients, *n* (%)	71,685
Total patients-days, *n* (%)	652,167
Average LOS, mean, SD	Mean = 7.09, SD = 7.66
Survival status, *n* (%)	
Alive	60,795 (84.81%)
Death	10,890 (15.19%)
Number of countries, stratified per income level according to World Bank	
Low-income country	1 (8.33%)
Lower middle-income country	1 (8.33%)
Upper middle income country	10 (83.33%)
Number of patients admitted per facility ownership, *n* (%)	
For-profit privately owned facilities	38,787 (54.11%)
Publicly owned facilities	20,880 (29.13%)
University	10,545 (14.71%)
Not-for-profit privately owned facilities	1,473 (2.05%)
Hospitalization type	
Number of patients with medical Hospitalization, *n* (%)	46,892 (65.41%)
Number of patients with surgical Hospitalization, *n* (%)	24,793 (34.59%)
Number of patients admitted per type of ICU, *n* (%)	
Cardio-thoracic ICU	1,372 (1.91%)
Coronary ICU	12,995 (18.13%)
Medical ICU	2,732 (3.81%)
Medical-surgical ICU	45,538 (63.52%)
Neuro-surgical ICU	655 (0.91%)
Neurologic ICU	101 (0.14%)
Adult oncology ICU	186 (0.26%)
Pediatric oncology ICU	13 (0.018%)
Pediatric ICU	4,457 (6.22%)
Respiratory ICU	560 (0.78%)
Surgical ICU	2,890 (4.03%)
Trauma ICU	186 (0.26%)
Gender, *n* (%)	
Male	39,063 (54.49%)
Female	32,622 (45.51%)
Age, mean, SD	Mean = 55.34, SD = 24.31
Device-days and device utilization ratio	
CL-days, *n*, mean, SD	348,609, mean = 4.86, SD = 9.47
MV-days, *n*, mean, SD	199,397, mean = 2.78, SD = 6.6
UC-days, *n*, mean, SD	315,348, mean = 4.40, SD = 6.62
CL-utilization ratio, mean, SD	245,181, mean = 3.42, SD = 6.19
MV-utilization ratio, mean, SD	Mean = 0.59, SD = 1.43
UC-utilization ratio, mean, SD	Mean = 0.29, SD = 0.88
Healthcare-associated infections	
CLABSI, *n* (%)	1,324 (28.17%)
VAP, *n* (%)	2,439 (51.89%)
CAUTI, *n* (%)	937 (19.94%)

*ICU* intensive care unit, *CL* central line, *MV* mechanical ventilator, *UC* urinary catheter, *LOS* length of stay, *CLABSI* central line associated bloodstream infection, *VAP* ventilator-associated pneumonia, *CAUTI* catheter associated urinary tract infection, *SD* standard deviation

Data on 71,685 critical patients was gathered and they were followed from admission to discharge from ICU during 652,167 patient-days, they acquired 4700 HAIs, and 10,890 died.

Rates of mortality, CLABSI, VAP and CAUTI stratified per country are shown in Table 2. Mortality rates stratified per ICU type are shown in Table 3. Mortality rates stratified per WB country classification by income level (lower-middle income, upper-middle income, and high income) and by facility ownership type (publicly owned facilities, for-profit privately owned facilities, university hospitals, not-for-profit privately owned facilities) are shown in Table 4.

**Table 2:** Mortality, ventilator-associated pneumonia, central line associated bloodstream infections, and catheter-associated urinary tract infections rates stratified per country

Country^a^	Patients, *n*	Dead patients, *n* (Mortality rate %), (95% CI)	CLABSI, *n* /CL-days, *n* = CLABI rate (95% CI)	MV-days, *n*/VAP, *n* = VAP rate (95% CI)	CAUTI, *n*/UC-days, *n* = CAUTI rate (95% CI)
1. Cuba	977	322 (32.96%)	3/3597 = 0.834	16/2,632 = 6.08	¾,686 = 0.64
		95% CI: (29.46–36.76)	95% CI: (0.80–0.86)	95% CI: (5.98–6.17)	95% CI: (0.61–0.66)
2. Ecuador	908	265 (29.19%)	30/8,481 = 3.54	226/3,844 = 58.79	59/6,204 = 9.51
		95% CI: (25.78–32.92)	95% CI: (3.49–3.57)	95% CI: (58.55–59.03)	95% CI: (9.43–9.58)
3. Argentina	22460	4011 (17.86%)	295/54,649 = 5.40	429/35,160 = 12.20	282/90,110 = 3.13
		95% CI: (17.31–18.42)	95% CI: (5.37–5.41)	95% CI: (12.16–12.23)	95% CI: (3.11–3.14)
4. Brazil	14945	2203 (14.74%)	148/110,601 = 1.34	488/54,325 = 8.98	183/62,749 = 2.92
		95% CI: (14.13–15.37)	95% CI: (1.33–1.34)	95% CI: (8.95–9.00)	95% CI: (2.90–2.93)
5. Panama	845	124 (14.67%)	86/7,600 = 11.32	36/6,064 = 5.94	38/6,779 = 5.61
		95% CI: (12.21–17.50)	95% CI: (11.24–11.39)	95% CI: (5.87–5.99)	95% CI: (5.54–5.66)
6. Dominican Republic	1396	200 (14.33%)	61/4,331 = 14.09	43/2,590 = 16.60	24/5,047 = 4.76
		95% CI: (12.41–16.46)	95% CI: (13.97–14.19)	95% CI: (16.44–16.76)	95% CI: (4.69–4.81)
7. Peru	1830	261 (14.26%)	16/6,763 = 2.37	47/4,209 = 11.17	10/7,121 = 1.40
		95% CI: (12.58–16.10)	95% CI: (2.32–2.40)	95% CI: (11.06–11.26)	95% CI: (1.37–1.43)
8. El Salvador	595	79 (13.28%)	21/3,078 = 6.82	38/3,305 = 11.50	5/2,498 = 2.00
		95% CI: (10.51–16.55)	95% CI: (6.73–6.91)	95% CI: (11.38–11.61)	95% CI: (1.94–2.05)
9. Mexico	8655	1112 (12.85%)	419/60,105 = 6.97	852/37,580 = 22.67	233/49,828 = 4.68
		95% CI: (12.10–13.63)	95% CI: (6.95–6.99)	95% CI: (22.62–22.72)	95% CI: (4.65–4.69)
10. Colombia	16418	2095 (12.76%)	213/80017 = 2.66	204/45091 = 4.52	85/72021 = 1.18
		95% CI: (12.22–13.32)	95% CI: (2.65–2.67)	95% CI: (4.50–4.54)	95% CI: (1.17–1.18)
11. Venezuela	1246	127 (10.19%)	29/4871 = 5.95	36/2718 = 13.25	15/4570 = 3.28
		95% CI: (8.50–12.13)	95% CI: (5.88–6.02)	95% CI: (13.10–13.38)	95% CI: (3.23–3.33)
12. Costa Rica	1410	91 (6.45%)	3/4516 = 0.66	24/1879 = 12.77	0/3735 = 0
		95% CI: (5.19–7.92)	95% CI: (3.23–3.33)	95% CI: (12.61–12.93)	95% CI: NA

*CL* central line, *MV* mechanical ventilator, *UC* urinary catheter, *CLABSI* central line-associated bloodstream infection, *VAP* ventilator-associated pneumonia, *CAUTI* catheter-associated urinary tract infection, *CI* confidence interval

^a^Countries are listed in order of the highest to lowest mortality rate

**Table 3:** Mortality rates stratified per ICU type

ICU type^a^	Patients, *n*	Dead patients, *n* (mortality rate %), (95% CI)
Respiratory ICU	560	177 (31.61%) 95% CI: (27.12–36.62)
Neurologic ICU	101	25 (24.75%) 95% CI: (16.02 –36.54)
Neuro-surgical ICU	655	142 (21.68%) 95% CI: (18.26–25.55)
Medical-surgical ICU	45,538	8309 (18.25%) 95% CI: (17.86–18.64)
Adult-oncology ICU	186	30 (16.13%) 95% CI: (10.88–23.03)
Medical ICU	2732	354 (12.96%) 95% CI: (11.64–14.38)
Cardio-thoracic ICU	1372	144 (10.5%) 95% CI: (8.85–12.36)
Trauma ICU	186	18 (9.68%) 95% CI: (5.74–15.29)
Pediatric ICU	4457	394 (8.84%) 95% CI: (7.99–9.75)
Coronary ICU	12,995	1080 (8.31%) 95% CI: (7.82–8.82)
Pediatric-oncology ICU	13	1 (7.69%) 95% CI: (0.19–4.28)
Surgical ICU	2890	216 (7.47%) 95% CI: (6.51–8.54)

*ICU* intensive care unit, *CI* confidence interval

^a^ICUs are listed in order of the highest to lowest mortality rate

**Table 4:** Mortality rate stratified per World Bank country classifications by income level and per facility ownership type

	Patients, *n*	Dead patients, *n* (mortality rate %), (95% CI)
Lower-middle income		
Publicly owned facilities	595	79 (13.28%) 95% CI: (10.51–16.55)
Upper-middle income		
Pooled	69,844	10,684 (15.3%) 95% CI: (15.01–15.59)
Publicly owned facilities	20,178	3,351 (16.61%) 95% CI: (16.05–17.10)
For-profit privately owned facilities	37,648	5,766 (15.32%) 95% CI: (14.92–15.72)
University hospitals	10,545	1,336 (12.67%) 95% CI: (12.00–13.37)
Not-for-profit privately owned facilities	1473	231 (15.68%) 95% CI: (13.73–17.84)

*CI* confidence interval

Using multiple logistic regression, the following eleven variables were identified as RF statistically significantly independently associated with “in ICU all cause-mortality” (Table 5): (1) VAP acquisition; (2) CAUTI acquisition; (3) older age rising the risk 2% per year; (4) longer indwelling time of CL-days, rising the risk 3% per CL-day; (5) longer indwelling time of UC-days, rising the risk 1% per UC-day; (6) higher MV-utilization ratio; (7) higher UC-utilization ratio; (8) for-profit privately owned facilities and Publicly owned facilities, compared with University hospitals; (9) medical hospitalization instead of surgical; (10) lower middle income countries; and (11) ICUs with the highest associated risk for mortality, ranked from highest to lowest, were neurologic (RR = 4.48; *p* < 0.0001), adult oncology (RR = 3.48; *p* < 0.0001), neuro surgical (RR = 2.49; *p* < 0.0001), medical surgical (RR = 2.06; *p* < 0.0001), respiratory (RR = 1.96; *p* < 0.0001), pediatric (RR = 1.67; *p* < 0.0001) and medical (RR = 1.44; *p* = 0.001).

**Table 5:** Multiple logistic regression analysis of risk factors for mortality

	aOR	95% CI	*P* value
CLABSI	1.09	0.95–1.26	0.20
VAP	1.17	1.06–1.30	< 0.0001
CAUTI	1.34	1.15–1.56	< 0.0001
Age	1.02	1.01–1.02	< 0.0001
Gender, male	1.01	0.97–1.06	0.57
Length of stay	1.01	0.99–1.01	0.58
CL-days,	1.03	1.02–1.03	< 0.0001
MV-days	0.98	0.97–0.99	< 0.0001
UC-days	1.01	1.01–1.27	< 0.0001
CL-utilization ratio	0.81	0.78–0.85	< 0.0001
MV-utilization ratio	6.47	5.96–7.03	< 0.0001
UC-utilization ratio	1.19	1.11–1.26	< 0.0001
For-profit privately owned facilities	1.50	1.27–1.77	< 0.0001
Publicly owned facilities	1.47	1.24–1.74	< 0.0001
University hospital	1.09	0.91–1.30	0.32
Lower middle income country	2.94	2.10–4.12	< 0.0001
Upper middle income country	2.04	1.67–2.48	< 0.0001
Medical hospitalization	1.67	1.59–1.75	< 0.0001
Neurologic ICU	4.48	2.68–7.50	< 0.0001
Adult-oncology ICU	3.48	2.14–5.65	< 0.0001
Neuro-surgical ICU	2.49	1.89–3.29	< 0.0001
Medical-surgical ICU	2.06	1.70–2.49	< 0.0001
Respiratory ICU	1.96	1.49–2.57	< 0.0001
Pediatric ICU	1.67	1.32–2.11	< 0.0001
Medical ICU	1.44	1.15–1.81	0.001
Pediatric-oncology ICU	3.67	0.45–30.08	0.23
Coronary ICU	1.02	0.84–1.25	0.84
Surgical ICU	1.18	0.93–1.51	0.17
Trauma ICU	1.47	0.86–2.52	0.16

*ICU* intensive care unit, *CL* central line, *MV* mechanical ventilator, *UC* urinary catheter, *LOS *length of stay, *CLABSI* central line associated bloodstream infection, *VAP* ventilator-associated pneumonia, *CAUTI* catheter-associated urinary tract infection, *aOR* adjusted odds ratio, *CI* confidence interval

## Discussion

According to the literature, although device utilization ratio in Latin American ICUs was similar to that reported in CDC-NHSN ICUs [20], HAI rates are higher in Latin America [12–19].

The present study found an association between the VAP or CAUTI acquisition and mortality, and this is consistent with findings of Ylipalosaari et al. The authors found that in multivariate logistic regression analysis, ICU-acquired infection remained an independent mortality RF after adjustment for APACHE II score and age [30].

Older age was also found to be associated with mortality in our study. A 2% rise in mortality per year of age was observed in our study. Similarly in an analysis of mortality RF in a Turkish university hospital, with logistic regression analyses, age (> 60 years) was found to be significant mortality RF [31].

This study further observed an association between longer indwelling time of CL-days, longer indwelling time of urinary catheter days, and higher UC-utilization ratio and mortality. We found a 3% increase in mortality per day of use of CL and a 1% increase in mortality per day of use of UC. Showing a similar association in a study analyzing risk factors for mortality from VAP a study concluded that CL was associated with higher risks for all-cause mortality in an ICU [22].

Furthermore, this study found an association between higher MV-utilization ratio and mortality. In a study conducted by Kalin et al., the need for MV was also established to be independent mortality RF [32].

The current study also observed a significantly higher risk of mortality at for-profit privately owned facilities or publicly owned facilities compared with university hospitals. Analyzing this same association, Eggleston carried out a systematic review trying to explain what the findings are relating to the ownership and quality of hospitals. They found that ownership is systematically related to differences in quality between hospitals. Those studies found that for-profit, government-controlled hospitals have higher mortality rates than non-profit hospitals [33].

Additionally, the present study found that lower middle-income countries have a significantly higher risk of mortality. This result could be explained by considering that healthcare quality programs are probably inadequate in lower-middle-income countries.

In addition, this study found that medical hospitalization has a significantly higher risk of mortality than surgical hospitalization. This may be attributed to the fact that, on the one hand, patients admitted for planned surgical procedures typically have less severe conditions than those admitted for medical reasons and are therefore more stable [34].

The present study noted that patients admitted to neurologic, neuro-surgical, adult oncology, neuro surgical and medical surgical ICUs were associated with the highest risk of mortality than patients hospitalized in other kind of ICU.

Our study failed to find an association between gender and mortality. In contrast, an investigation in a surgical ICU that analyzed mortality RF in patients with CLABSI demonstrated with multivariate analysis that men have been shown to be more associated with death than women [21].

Our research also found no association between prolonged stay and mortality. This finding is inconsistent with a previous study looking at the epidemiology and mortality RF in VAP, in which prolonged hospital stay was noted to be an independent risk factor in the multivariate analysis. This finding of our study could be explained by the fact that patients in Latin America are often hospitalized for prolonged periods awaiting surgical interventions, and meanwhile they are stable without having invasive devices, such as CL or a MV [35].

Some of the mortality RFs identified in our study are unlikely to change, such as the income level of the country, facility ownership, hospitalization type, ICU type, and age. However, some of the mortality RFs we identified can be modified; for example, longer indwelling time of CL-days, longer indwelling time of UC-days, higher MV-utilization ratio as a marker of severity of illness of patients, higher UC-utilization ratio as a marker of severity of illness of patients, VAP acquisition, and CAUTI acquisition. As HAI rates in Latin America are significantly higher than in the US, there is room for improvement [12–19]. Based on our findings, it is suggested that we focus on strategies to reduce indwelling time of CL-days, UC-days, MV-utilization ratio, UC-utilization ratio, and implement an evidence-based set of HAI prevention recommendations, such as those recently published by IDSA/SHEA/APIC [36, 37]. Also, the high rate of HAIs prevalent in the Latin America [12–19] can be reduced by utilizing a strategy of monitoring compliance with recommendations and providing performance feedback to healthcare personnel, as demonstrated by INICC in several ICUs of Latin America [38–45].

Our research has some limitations. First, this study is not representative of all hospitals in Latin America since it is a surveillance system in which hospitals voluntarily join. Second, it is likely that the hospitals that participate in our surveillance system are the ones that have a better quality HAI surveillance and prevention program, and for this reason, the HAI rates in our study are likely to be lower than the HAI rates found in other hospitals not participating in our study. Finally, participating hospitals have not collected data on disease severity scores and underlying diseases, but instead we collected DU-utilization ratio as a marker of the severity of illness of the patients [28].

## Conclusions

The most important finding that our research provide us is that there are a large number of variables that are associated with the risk of death in ICUs. Unfortunately most of them cannot be modified. Therefore, since they cannot be modified, we cannot obtain any impact on these variables to reduce mortality. But, on the other hand, we have found some variables that are feasible to be modified, and they are indwelling time of CL-days, indwelling time of UC-days, MV-utilization ratio, UC-utilization ratio, and implement an evidence-based set of HAI prevention recommendations [36, 37]. HAIs in ICUs in Latin America are several times higher than those in high-income countries [12–19], and a greater effort should be made to reduce the rates of HAIs, and thus be able to reduce this high mortality rate.

## Data Availability

Data and material are available for review.

## Abbreviations

aOR: Adjusted odds ratio
APACHE: Acute physiology and chronic health evaluation
CAUTI: Catheter-associated urinary tract infection
CDC: Centers for diseases control and prevention
CL: Central line
CLABSI: Central line-associated bloodstream infection
HAI: Healthcare-associated infection
ICU: Intensive care unit
INICC: International nosocomial infection control consortium
LOS: Length of stay
MV: Mechanical ventilator
NHSN: National healthcare safety network
aOR: Adjusted odds ratio
SD: Standard deviation
UC: Urinary catheter
VAP: Ventilator-associated pneumonia
